# Validez de la aplicación móvil PODÓMETRO® en la evaluación de la capacidad cardiorrespiratoria y resistencia a la fatiga en rehabilitación cardiaca. Estudio piloto

**DOI:** 10.23938/ASSN.1078

**Published:** 2024-12-02

**Authors:** Miguel Gómez-Alguacil, Virginia Llanos-Ruíz, Leire Moreno-Galdós, María Madrigal-Azcona, Antonio López-Román, Roberto Cano-de-la-Cuerda, Isabel M Alguacil-Diego

**Affiliations:** 1 Universidad Alfonso X El Sabio Facultad de Medicina y de Ciencias de la Salud Villanueva de la Cañada Madrid España; 2 HM Hospitales. Hospital Universitario HM Puerta del Sur Servicio de Rehabilitación Unidad de Rehabilitación Cardiaca Móstoles Madrid España; 3 Fundación de Investigación HM Hospitales Madrid Madrid España; 4 HM Hospitales. Hospital Universitario HM Puerta del Sur Servicio de Cardiología Móstoles Madrid España; 5 HM Hospitales. Hospital Universitario HM Montepríncipe Unidad de Rehabilitación Cardiaca Boadilla del Monte Madrid España; 6 HM Hospitales. Hospital Universitario HM Puerta del Sur Servicio de Fisioterapia Móstoles Madrid España; 7 Universidad Rey Juan Carlos Departamento de Fisioterapia, Terapia Ocupacional, Rehabilitación y Medicina Física Alcorcón Madrid España; 8 HM Hospitales. Hospital Universitario HM Puerta del Sur Servicio de Rehabilitación Móstoles Madrid España

**Keywords:** Aplicaciones Móviles, Capacidad Cardiorrespiratoria, Rehabilitación Cardiaca, Test de Marcha de los 6 Minutos, Cardiac rehabilitation, Cardiorespiratory capacity, Mobile applications, 6 Minutes Walking Test

## Abstract

**Fundamento:**

Una aplicación móvil (*app*) podría facilitar la realización del test de marcha de los 6 minutos (TM6M), frecuentemente utilizado en rehabilitación cardiaca. El objetivo de este estudio piloto es estudiar en pacientes en rehabilitación cardiaca 1) la validez de criterio de la *app* PODÓMETRO^®^ frente al TM6M en la evaluación de la capacidad cardiorrespiratoria y resistencia a la fatiga y 2) la validez de constructo de la *app* PODÓMETRO^®^ con la Escala Hospitalaria de Ansiedad y Depresión (HADS) y con el índice cardinal de salud del cuestionario EuroQol-5D 3L (EQ-5D).

**Método:**

Veinte pacientes en rehabilitación cardiaca en el Hospital HM Puerta del Sur (Móstoles, España) realizaron el TM6M con la *app* PODÓMETRO^®^ instalada en un *smartphone* en el brazo. Se calculó el coeficiente de correlación de Pearson entre la distancia registrada en metros durante el TM6M y por la *app*, así como entre la *app* y las dimensiones ansiedad y depresión de la HADS, y el índice cardinal de salud EQ-5D.

**Resultados:**

La validez de criterio entre el TM6M y la *app* PODÓMETRO^®^ fue excelente (r = 0,981; p<0,001). Respecto a la validez de constructo, se observó una correlación negativa y moderada (r = -0,493) entre la *app* y la dimensión depresión de la HADS, pero no con la dimensión ansiedad ni con el EQ-5D.

**Conclusiones:**

La *app* gratuita PODÓMETRO^®^ presenta una excelente validez de criterio al compararse con el TM6M. La *app* solo se correlacionó con la variable depresión de la HADS, de forma negativa y moderada.

## Abstract (en)

**Background:**

A mobile application (app) may facilitate the administration of the 6-minute walk test (6MWT), a commonly used tool in cardiac rehabilitation. This pilot study, conducted with cardiac rehabilitation patients, aimed to evaluate: 1) the criterion validity of the PEDOMETER^®^ app compared to the 6MWT in assessing cardiorespiratory fitness and fatigue resistance, and 2) the construct validity of the PEDOMETER^®^ app in relation to the Hospital Anxiety and Depression Scale (HADS) and the Health Index of the EuroQol-5D 3L questionnaire (EQ-5D).

**Methods:**

Twenty patients undergoing cardiac rehabilitation at HM Puerta del Sur Hospital (Mostoles, Spain) performed the 6MWT while using the PEDOMETER^®^ app on a smartphone worn on their arm. Pearson’s correlation coefficient was calculated to assess the relationship between the distance recorded in meters during the 6MWT and that recorded by the app, as well as between the app and the anxiety and depression dimensions of the HADS, and the Health Index EQ-5D.

**Results:**

The criterion validity of the 6MWT and the PEDOMETER^®^ app was excellent (r = 0.981; p<0.001). Regarding construct validity, the app showed a moderate negative correlation with the depression dimension of the HADS (r = -0.493), but not with the anxiety dimension or the EQ-5D.

**Conclusions:**

The free PEDOMETER^®^ app demonstrates excellent criterion validity when compared to the 6MWT. The app shows a mode-rate negative correlation with the depression dimension of the HADS.

## INTRODUCCIÓN

Las aplicaciones móviles o *apps* dedicadas al área sanitaria aportan una nueva visión del cuidado de la salud, tanto para profesionales sanitarios como para pacientes^1^^-^^3^, aunque seleccionarlas según criterios de validez y fiabilidad resulta complejo^4^^,^^5^. Por ello, y ante la proliferación de estas *apps*, se están realizando labores de regulación y denominación de criterios de calidad mínimos^6^^-^^9^.

En el ámbito de la rehabilitación cardiaca (RC) se realiza habitualmente el test de marcha de los seis minutos^10^ (TM6M), un instrumento eficaz y seguro en la evaluación del estado funcional, la eficacia terapéutica, la orientación de modalidades de entrenamiento, y el establecimiento del pronóstico de morbimortalidad^11^^-^^13^. Este test se considera una herramienta segura, de bajo coste y reproducible, que está validado para evaluar, a una intensidad submáxima, la distancia en metros que el paciente es capaz de recorrer durante un tiempo de seis minutos. Distancias inferiores a 300 m indican una mayor probabilidad de hospitalizaciones con un mayor riesgo de muerte, y velocidades inferiores a 4,4 km/h son predictoras de alta probabilidad de caídas, dependencia y mal pronóstico de supervivencia^11^^,^^12^. No obstante, su aplicación implica tiempo, supervisión y un entorno que requiere una preparación previa, por lo que el uso de una *app* móvil podría permitir condiciones de automonitorización y seguimiento en tiempo real en los programas de rehabilitación cardiaca, de forma cómoda, sencilla y económica. 

Estudios previos han validado *apps* de geolocalización en otras patologías con excelente validez de criterio al compararlas con pruebas de marcha^14^^,^^15^, mostrándolas como un complemento a los métodos convencionales de análisis de la marcha en la práctica clínica. 

El estado de ánimo se asocia tanto al desarrollo como a la progresión de la enfermedad cardiovascular, y las emociones *negativas* se han asociado a un aumento de las tasas de muerte cardiovascular y a eventos cardiacos recurrentes, aunque continúan sin estar claros los mecanismos que explican esta asociación^13^. Factores psicológicos como la depresión y la ansiedad se asocian con una velocidad de marcha lenta^13^, y los síntomas de depresión predicen la incidencia del deterioro de la velocidad de marcha y viceversa^16^. Se ha observado relación bidireccional entre el estado físico y los factores psicológicos. Mientras la ansiedad, la depresión y la baja calidad de vida son factores limitantes tanto en la adhesión al programa de rehabilitación cardiaca hospitalario como en su cumplimiento una vez finalizada la fase hospitalaria^17^, la rehabilitación cardiaca mediante ejercicio físico favorece el aumento de la capacidad funcional, la calidad de vida y el estado psicológico^18^. Completar el programa de rehabilitación cardiaca se asocia a una reducción significativa del malestar psicológico derivado de la situación clínica^19^, aunque este efecto parece depender de la gravedad del estado psicológico, ya que una rehabilitación cardiaca temprana mejora el estado de ansiedad en pacientes con síntomas ansioso-depresivos leves^20^, pero no graves. Se han propuesto varios mecanismos fisiopatológicos para explicar estas relaciones, incluida la desregulación del eje hipotálamo-hipofisario-adrenal, la activación plaquetaria y la inflamación. También se han implicado factores conductuales, como la falta de adherencia a las terapias médicas prescritas y la inactividad física^21^.

Lo expuesto induce a considerar que la distancia recorrida, la ansiedad, la depresión y la calidad de vida relacionada con la salud (CVRS) puedan ser constructos interrelacionados y, por tanto, es necesario considerar estos factores en los procesos de validación de las pruebas de capacidad funcional en RC. 

Por tanto, el objetivo del presente estudio piloto es estudiar la validez de criterio de la aplicación móvil PODÓMETRO^®^ en la evaluación de la capacidad cardiorrespiratoria y resistencia a la fatiga en pacientes que realizan RC. Secundariamente, se pretende estudiar la validez de constructo entre la *app* y los niveles de ansiedad, depresión y CVRS, a través de la Escala Hospitalaria de Ansiedad y Depresión (HADS) y el cuestionario EuroQol-5D 3L (EQ-5D), respectivamente. 

## MÉTODO

### Diseño

Estudio transversal realizado en pacientes del Servicio de Cardiología del Hospital HM Puerta del Sur (Móstoles, España) derivados al Servicio de Rehabilitación, con prescripción de RC, entre los meses de enero y mayo de 2023. 

Se realizó un muestreo no probabilístico de casos consecutivos, siguiendo la guía STROBE para estudios observacionales^22^. Los criterios de inclusión establecidos por el Servicio de Cardiología para participar en un programa de rehabilitación cardiaca son: pacientes con cardiopatía isquémica, valvulopatías intervenidas, cardiopatías congénitas operadas, trasplante cardíaco, insuficiencia cardíaca, marcapasos o desfibriladores, personas sanas en riesgo cardiovascular o con desacondicionamiento físico. Los criterios de exclusión para participar en este estudio fueron: ser menor de edad, presentar un bajo nivel cognitivo, y personas diagnosticadas con otras enfermedades o condiciones que afecten a la capacidad de marcha o con alteraciones visuales graves.

### Procedimiento 

Se invitó a participar a personas que, derivadas del Servicio de Cardiología, estaban pendientes de iniciar RC, accediendo a la lista de espera de la Unidad de Rehabilitación Cardiaca del Servicio de Rehabilitación. Para participar en el estudio, cada participante tuvo que aceptar y firmar un consentimiento informado. Se siguieron los principios éticos para la investigación médica en humanos de la Declaración de Helsinki y se obtuvo la aprobación del Comité Ético del Hospital HM Puerta del Sur (Móstoles, España) (código 22.12.2121-GHM). 

Todas las valoraciones fueron realizadas por un mismo médico rehabilitador en el Servicio de Rehabilitación del Hospital HM Puerta del Sur (Móstoles, España), en similares condiciones ambientales. 

En la primera valoración se recogieron las características sociodemográficas y clínicas de los participantes: edad (años), sexo (mujer, hombre), altura (m), peso (kg), índice de masa corporal (IMC; kg/m^2^), síndrome coronario agudo (con o sin elevación del segmento ST), extensión (uno, dos o tres vasos afectados) y fracción de eyección del ventrículo izquierdo (FEVI; %); se considera un FEVI conservado cuando es ≥50%. Además, se calculó la frecuencia cardiaca máxima teórica mediante la fórmula: 220 - edad (años). 

A continuación, el paciente cumplimentó dos instrumentos:

- La escala HADS presenta propiedades psicométricas adecuadas para detectar posibles casos de ansiedad y depresión, habiendo sido validada en pacientes con cardiopatía^23^. El periodo examinado corresponde a los últimos siete días. Consta de 14 ítems con cuatro posibles respuestas (de 0 a 3) que consideran dimensiones cognitivas y afectivas, omitiendo aspectos somáticos que pudieran ser atribuidos a la enfermedad que presente la persona. Puntuaciones de 0 a 7 se consideran normales, de 8 a 10 dudosas, y ≥11 problema clínico.
- El EQ-5D es un cuestionario genérico de CVRS que ha probado su utilidad como medida de salud^24^. Consta de dos partes: 1) el sistema descriptivo EQ-5D que considera cinco dimensiones: movilidad, autocuidado, actividades habituales, dolor o malestar, y ansiedad o depresión), y que genera un índice cardinal de salud que oscila entre 1 (mejor estado de salud) y 0 (la muerte), aunque existen valores negativos correspondientes a aquellos estados de salud que son valorados como peores que la muerte; 2) la Escala Analógica Visual (EAV) donde la persona puntúa cómo percibe su salud el mismo día que cumplimenta el cuestionario, en un rango de 0 (peor estado de salud imaginable) a 100 (mejor estado de salud imaginable). Para este estudio se utilizó la versión 3L, que ofrece tres niveles de opciones de respuesta (ausencia de problema, algún problema, y problema extremo).

Todos los participantes realizaron el TM6M portando un teléfono inteligente (modelo iPhone 11) con la aplicación gratuita PODÓMETRO^®^, previamente descargada e instalada en el dispositivo. Si bien la *app* es de libre acceso, se solicitó y obtuvo el permiso correspondiente para su uso con fines científicos a la compañía propietaria (ITO Technologies, Inc, Kanagawa, Japón). 

**Figura 1:**
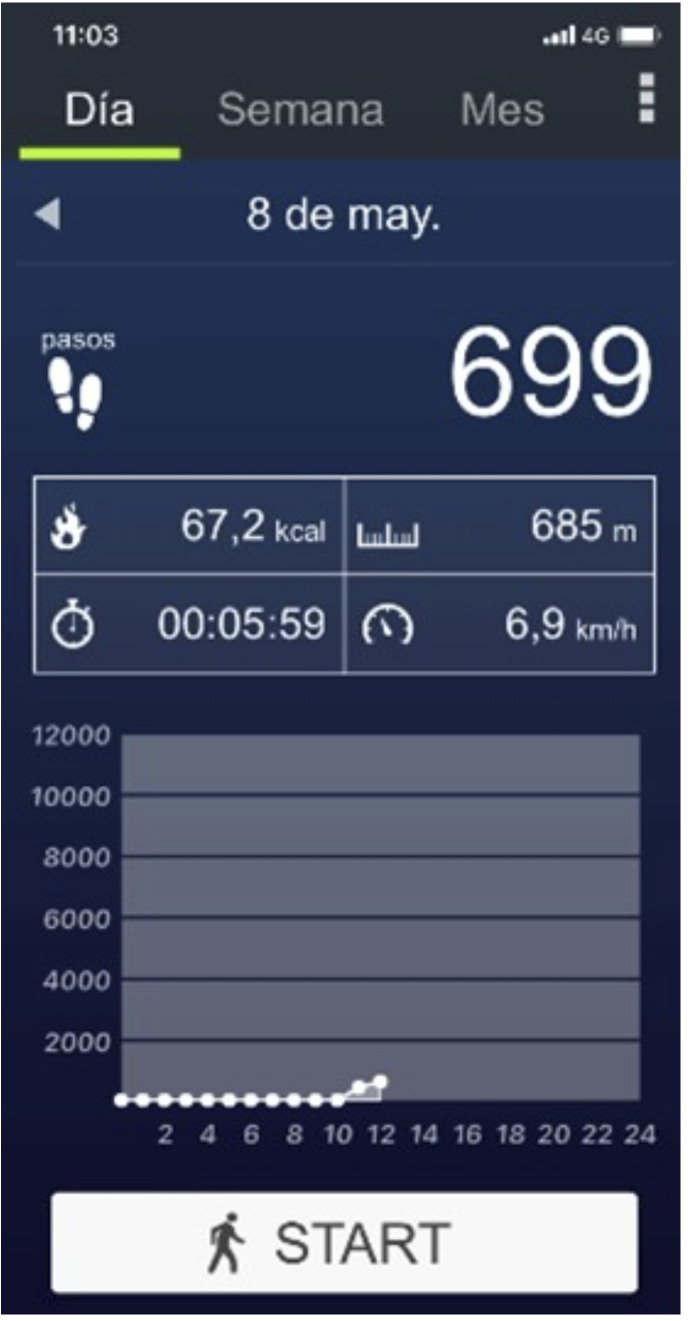
Aplicación móvil PODÓMETRO^®^ (ITO Technologies, Inc. Kanagawa, Japón).

PODÓMETRO^®^ está disponible en español para móviles con sistema operativo iOS y Android. Utiliza los datos del acelerómetro y del sistema de posicionamiento global del teléfono para medir los parámetros de marcha. Informa del número de pasos, la distancia recorrida en metros, la velocidad (km/h), el tiempo caminando en minutos y el consumo calórico (kcal) (Fig. 1). Este estudio solo analizó la distancia recorrida, para lo que fue necesario calcular e introducir la longitud de paso en metros (distancia recorrida en metros en 100 pasos en línea recta dividida entre 100). El dispositivo móvil iba sujeto a la extremidad superior del participante. Cuando el paciente empieza a caminar a la orden dada por el investigador, solo ha de presionar el botón inicio. Transcurridos los seis minutos, el investigador avisa y el paciente detiene la marcha y presiona el botón de parada.

**Figura 2:**
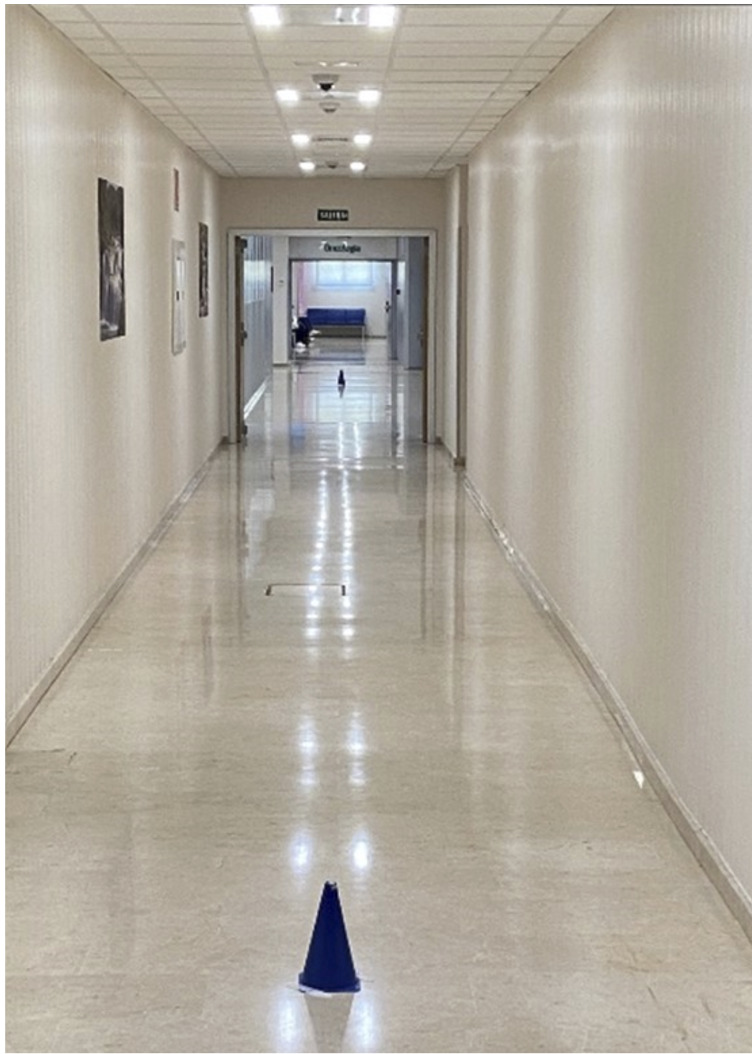
Pasillo donde se realizó el test de la marcha de los seis minutos, con los conos señalando los puntos de giro.

Mientras los participantes tenían activada la *app* PODÓMETRO^®^, se aplicó el protocolo del TM6M, aceptado por la SEPAR (Sociedad Española de Neumología y Cirugía torácica) siguiendo las recomendaciones de la Sociedad Americana de Tórax^10^^-^^14^^,^^16^^,^^17^^,^^25^^,^^26^. Se realizó en un pasillo de 30 metros de longitud ubicado en el interior del hospital. Cada paciente debía recorrer ese tramo, ida y vuelta, pudiendo parar si lo necesitaba, aunque el reloj no parase. Los puntos donde tenían que girar se señalizaron mediante dos conos separados entre sí 29 metros (Fig. 2). Todos los participantes caminaron supervisados por el examinador y se incentivó al paciente durante la realización de la prueba^25^. Por lo general, las personas sanas pueden caminar entre 400 y 700 m en 6 minutos, dependiendo de la edad, estatura y sexo. Sin embargo, el TM6M se ve influido no solo por la capacidad cardio-respiratoria del sujeto, sino también por factores antropométricos, músculo-esqueléticos y motivacionales^10^^,^^12^.

Se evaluó la percepción del esfuerzo mediante la escala de Borg modificada^27^ y la frecuencia cardiaca (FC) con un cardio-frecuenciómetro inalámbrico Ergoline^®^ (Bitz, Alemania)^28^. Además, se determinó la frecuencia cardíaca máxima alcanzada y el porcentaje de la frecuencia cardiaca máxima teórica alcanzada. Para considerar que la frecuencia cardíaca máxima alcanzada se encontraba en valores submáximos, el rango debía situarse en un rango producto de multiplicar la frecuencia cardiaca máxima teórica por 0,5 y por 0,85^12^.

### Análisis estadístico

Las variables cuantitativas se describieron mediante media y desviación estándar (DE) y las cualitativas mediante frecuencia absoluta y porcentaje. Las comparaciones por sexo se realizaron mediante t de Student. Los datos obtenidos por el TM6M y por la *app* PODÓMETRO^®^ se analizaron estadísticamente para determinar la validez de criterio de la *app* mediante el coeficiente de correlación de Pearson (r); la correlación se consideró débil si r <0,30, media-alta si 0,3 ≤ r ≤0,7 y muy alta si r >0,70^29^. El mismo coeficiente fue utilizado para estudiar la validez de constructo de la *app* PODÓMETRO^®^ con la HADS y con el índice cardinal de salud EQ-5D. Los datos se analizaron utilizando la versión 25.0 de SPSS^®^ (IBM). La significación estadística se situó en p < 0,05. 

## RESULTADOS

De los 25 pacientes pendientes de iniciar la rehabilitación cardiaca durante el periodo de estudio, 20 participantes cumplían los criterios de inclusión del estudio, aceptando participar en el mismo. 

Las características sociodemográficas y clínicas de las personas participantes se muestran en la tabla 1. La edad media fue 64,8 años (rango: 39 a 80), la mayoría eran hombres (n = 15; 75%), con sobrepeso (n = 9; 45%) u obesidad (n = 6; 30%). 

Trece pacientes (65%) había sufrido un síndrome coronario agudo sin elevación del segmento ST; 11 (55%) presentaban enfermedad severa con afectación de un solo vaso, cinco (25%) de tres vasos y 4 (20%) de dos, siendo el vaso más frecuentemente afectado la arteria coronaria derecha. Todos los pacientes fueron sometidos a revascularización, a excepción de una paciente con riesgo quirúrgico elevado. Solo cinco pacientes (25%) presentaban una FEVI reducida. No hubo diferencias por sexo excepto para edad, altura y calidad de vida (las mujeres tuvieron significativamente mayor edad, menor altura y menor calidad de vida).

**Tabla 1:** Características clínicas de la muestra

Característica	Global	Hombre	Mujer	p
	Media (DE)	Media (DE)	Media (DE)	t-Student
Edad (años)	64,8 (12,37)	62,13 (12,74)	72,8 (7,22)	<0,001
Altura (m)	1,72 (0,08)	1,75 (0,06)	1,62 (0,05)	<0,001
Peso (kg)	83,46 (20,44)	88,49 (20,85)	68,38 (9,15)	0,054
IMC (kg/m^2^)	27,99 (4,84)	28,64 (5,31)	26,04 (2,48)	0,311
**Puntuación HADS**				
Depresión	3,45 (2,65)	3 (2,42)	4,8 (3,11)	0,656
Ansiedad	4,05 (3,07)	3,87 (3,11)	4,6 (3,21)	0,195
Puntuación EQ5D_3L	0,92 (0,09)	0,95 (0,05)	0,83 (0,11)	0,002
Puntuación EAV	74 (11,42)	75 (10,69)	71 (14,32)	0,513

DE: desviación estándar; EAV: escala analógica visual del estado general de salud; EQ5D_3L: cuestionario de CVRS EuroQoL-5D con 3 niveles de respuesta; IMC: índice de masa corporal; HADS: Escala Hospitalaria de Ansiedad y Depresión.

La media de FC máxima alcanzada por los participantes en ambas sesiones (79,8 lpm; DE = 11,80) fue muy inferior la teórica (155,2 lpm; DE = 12,37), suponiendo el 51,68% de la misma (DE = 8,33), lo que identifica al 6MMT como una prueba submáxima. La percepción del esfuerzo realizado mediante la escala de Borg modificada (media 2,5; DE = 0,76) lo identificó entre muy ligero y ligero. 

La distancia media recorrida en el TM6M fue 508,6 m (DE = 107,17), valor situado en la horquilla de la población sana sin normalizar. La distancia media recogida en la *app* fue 500,91 m (DE = 97,11) y fue significativamente mayor en hombres que en mujeres (Tabla 2).

**Tabla 2:** Distancia en metros recorrida en el 6MMT y registrada con la aplicación móvil PODÓMETRO^®^

	Global	Hombre	Mujer	p
	Media (DE)	Media (DE)	Media (DE)	t-Student
**Distancia recorrida (m)**				
TM6M	508,62 (107,17)	532,44 (111,76)	437,14 (72,05)	0,129
PODÓMETRO®	500,91 (97,11)	522,70 (101,49)	435,56 (63,09)	0,035

DE: desviación estándar; TM6M: test de marcha de los 6 minutos.

El coeficiente de correlación mostró una validez de criterio excelente entre la distancia en metros recogida en el TM6M y la registrada con la *app* (r = 0,981; p<0,001).

Ningún paciente mostró en la HADS un problema clínico (con puntuaciones <11) y el EQ-5D mostró un índice medio de 0,92, ambos sin correlación estadística con la distancia recorrida en metros registrada con la *app* (r = -0,270; p = 0,249). Solo para la dimensión Depresión de la HADS se encontró una correlación negativa (r = -0,493) y estadísticamente significativa (p = 0,027) con la distancia registrada con la *app*. 

## DISCUSIÓN

En nuestro conocimiento, este estudio piloto es el primero que ha explorado la validez de criterio de una aplicación móvil gratuita (PODÓMETRO^®^) como alternativa al TM6M en pacientes subsidiarios de RC, así como su relación con variables psicológicas y de CVRS. 

Este estudio ha mostrado una correlación significativa entre TM6M evaluado de forma convencional y la *app*, lo que indica su validez de criterio en pacientes con patología cardiaca. El coeficiente de correlación fue superior a 0,80, por lo que se interpretó como excelente. 

Sin embargo, aunque PODÓMETRO^®^ se correlacionó de manera moderada-alta con la dimensión depresión de la HADS, no se relacionó con el distrés psicológico, lo que podría deberse a que la escala HADS mide el distrés psicológico presentado en la última semana, pudiéndose no correlacionarse con la capacidad aeróbica mostrada por el paciente el día de la valoración. Tampoco no se correlacionó con el índice cardinal de salud EQ-5D, lo que podría deberse, además de al reducido tamaño de la muestra, al efecto techo que presenta el cuestionario o a que, al ser un instrumento genérico de CVRS, su capacidad discriminativa y sensibilidad sean inferiores respecto de un instrumento específico. 

La incidencia de trastornos psicológicos en pacientes que han sufrido un infarto de miocardio oscila entre el 37 y el 58%. La depresión o la ansiedad son considerados factores de riesgo de la cardiopatía isquémica, ya que la depresión puede duplicar el riesgo de mortalidad después de un infarto de miocardio^21^^,^^23^^,^^30^. La ansiedad se ha asociado de forma menos consistente a un aumento de la mortalidad cardiaca, ya que las observaciones al respecto parecen depender en parte de la gravedad de la ECV^30^. Además, las reacciones psicológicas de negación de la enfermedad pueden dificultar la colaboración de la persona enferma, necesaria para el éxito del programa de RC. El tratamiento de estos procesos mejorará la calidad de vida a corto y largo plazo. PODÓMETRO^®^ se relacionó con la dimensión depresión de la HADS en pacientes que iban a comenzar la RC. No hemos encontrado estudios previos mediante el empleo y validación de aplicaciones móviles con los que poder comparar nuestros hallazgos. Nuestra hipótesis apuntaría a que los factores psicológicos, como la depresión, podrían asociarse a una disminución en la velocidad de marcha y, por tanto, en el número de pasos y en la distancia recorrida en el paciente con cardiopatía. Previamente, se ha estudiado que las personas con depresión muestran anomalías en la postura, el equilibrio y la marcha^31^, por lo que podría existir una relación incluso más clara en combinación con un proceso cardíaco. Futuros estudios deberían profundizar en dicha asociación.

Respecto a la respuesta cronotrópica, los valores submáximos alcanzados en el 6MMT fueron inferiores a los valores submáximos observados por González y col^12^ en su muestra de pacientes con cardiopatía sometidos a RC. Que nuestra muestra no haya alcanzado un mayor porcentaje de frecuencia cardíaca máxima ha podido estar motivado por que nuestros pacientes aún no habían comenzado el programa de RC y por que no todos los participantes se esforzasen al máximo al realizar la prueba, a pesar de haberles animado a ello siguiendo las recomendaciones de la SEPAR.

La literatura científica recoge estudios en los que se compara el TM6M con aplicaciones móviles en sujetos sanos o en poblaciones con trastornos específicos. Así, Jesus y col^32^ evaluaron la validez de criterio de la *app* Pacer^®^ y el TM6M en adultos sanos, tanto en interior como en exterior. Si bien la fiabilidad test-retest fue buena, la validez de constructo observada no alcanzó la significación. Cano-de la Cuerda y col^15^, estudiaron la validez de la app RUNZI^®^ para evaluar la capacidad aeróbica en sujetos con síndrome post-COVID, observando que la distancia en metros medida con la *app* mostró una excelente correlación con el TM6M. Salvi y col^33^ estudiaron la precisión de una *app* para evaluar el TM6M en un entorno clínico en personas con hipertensión arterial pulmonar, en comparación con un entorno al aire libre; su *app* mostró una excelente fiabilidad al aire libre. En nuestro caso, el procedimiento se llevó a cabo en una planta semisótano del centro hospitalario, sin detectarse problema alguno con la captación de la señal. Parece razonable tener en consideración la cobertura telefónica del dispositivo a emplear, así como su capacidad para detectar las señales. 

En el campo de la cardiología se han investigado múltiples *apps*: VascTrac no pudo medir con precisión la distancia realizada durante el TM6M en personas con enfermedad arterial periférica^34^, pero su uso para evaluar el TM6M en personas con ECV en domicilio y en un entorno clínico sugirió que puede monitorear su fragilidad y capacidad funcional en forma remota de manera segura y precisa^35^. Ziegl y col^36^ desarrollaron una *app* TM6M para pacientes con insuficiencia cardíaca, con resultados dispares según la localización del dispositivo móvil, y Scherrenberg y col^37^ desarrollaron otra *app* que permitía al paciente con cardiopatía autoadministrarse el TM6M en su domicilio en un trayecto aleatorio, resultando una medida fiable para la medición de su capacidad aeróbica. También la *app* gratuita PODÓMETRO^®^, situada en el brazo del participante, ha mostrado ser una herramienta útil, en ambientes cerrados, por lo que sería interesante valorar la capacidad de monitorización de la *app* como herramienta de telerrehabilitación en domicilio.

La adopción en masa de teléfonos inteligentes plantea su posible utilidad y validación como herramienta clínica. Futuras investigaciones deberían estudiar la capacidad de PODÓMETRO^®^ para automonitorizar pacientes, lo que podría ser un factor que aumentase la adhesión a los programas de RC. Igualmente, sería interesante comprobar su capacidad de *empoderar* al paciente -capacitarlo para tomar decisiones y ejercer control sobre su vida personal-, esencial en la promoción de la salud. También es necesario conocer la sensibilidad de la *app* al cambio clínico, y analizar otra información proporcionada como velocidad, cadencia de la marcha o consumo energético, y estudiar su relación con otros constructos (como actividad física o función ventricular). Hasta ahora, la literatura científica solo ha mostrado resultados en muestras mayoritariamente masculinas^34^^-^^37^ y con edades medias relativamente similares a la observada en este estudio^34^^,^^35^^,^^37^, por lo que sería necesario investigar el impacto del sexo en los resultados obtenidos con la *app* en pacientes con cardiopatía. Igualmente, puede resultar interesante conocer la correlación entre la *app* PODÓMETRO^®^ y el test TM6M en población más joven.

Este trabajo no está exento de limitaciones. Primera: se ha descrito un efecto aprendizaje en el TM6M, proponiéndose realizar dos pruebas seguidas con unos minutos de descanso entre ellas y escoger la mejor^38^. Al tratarse de pacientes con cardiopatía que a continuación debían realizar su sesión de RC, se decidió no seguir esta recomendación; además, tuvimos en cuenta que la reproducibilidad es mayor cuando la prueba se realiza siempre a la misma hora del día^38^. Segunda: si bien es cierto que el EQ-5D 3L presenta un efecto techo y menor poder discriminativo que la versión 5L, los resultados de su correlación con la *app* no hubiesen diferido. Tercera: la sintomatología de ansiedad y depresión se evaluaron de forma separada, cuando ambas están fuertemente correlacionadas en pacientes con cardiopatía; dado que los pacientes evalúan la sintomatología de ansiedad y depresión como un grupo de síntomas indistintos, se propuso que la estructura factorial de la HADS se evalúe de forma global en este grupo de personas^26^. Cuarta: el TM6M implica un ejercicio submáximo que se asemeja a los esfuerzos realizados en la vida cotidiana del paciente con enfermedad coronaria, pero la gravedad de la enfermedad parece influir en la intensidad de realización de la prueba^38^. Sin embargo, en el diseño del estudio no se tuvieron en consideración parámetros relacionados con la función cardiaca porque cada paciente es su propio control. 

En conclusión, los resultados del presente estudio piloto muestran que la aplicación móvil gratuita PODÓMETRO^®^ presenta una excelente validez de criterio en comparación con el TM6M. Sin embargo, no se relaciona ni con la ansiedad medida con el HADS ni con la CVRS evaluada con el EQ-5D, y solo muestra una correlación negativa moderada con la depresión medida con el HADS. Si bien se trata de un estudio de pilotaje de la *app*, esta podría ser una alternativa de interés al test TM6M convencional en pacientes con cardiopatía subsidiarios de rehabilitación cardiaca. Son necesarios futuros estudios con un mayor tamaño muestral y en poblaciones con otro tipo de patología, ya que las características especiales de la muestra no permiten extrapolar los resultados.

## Data Availability

Los datos del estudio se adjuntan como material suplementario.
